# Recurrent Ileal Variceal Bleeding as a Diagnostic and Therapeutic Challenge

**DOI:** 10.1155/2022/7072961

**Published:** 2022-04-26

**Authors:** Marek Cingel, Jakub Benko, Matej Samoš, Marián Mokáň

**Affiliations:** Clinic of Internal Medicine I of University Hospital Martin, Jessenius Faculty of Medicine, Comenius University, Bratislava, Slovakia

## Abstract

**Introduction:**

Massive ileal variceal bleeding is a rare intricate condition that needs rapid management and treatment. The absence of randomized clinical trials in this field leads to a lack of evidence-based diagnostic and therapeutical approaches. We present a case report describing imaging, endoscopic, and surgical procedures leading to the diagnosis and resolution of severe ileal variceal bleeding. *Case Report.* We admitted a 63-year-old patient for recurrent anemia and ongoing bleeding from the gastrointestinal tract presenting as enterorrhagia. We were not able to elucidate the source by endoscopic, angiographic, or nuclear imaging methods. As a last resort, we carried out a surgical procedure with peroperative enteroscopy and subsequent resection of the affected part of the intestine.

**Conclusion:**

We present a patient with a case of ileal variceal bleeding, which required extensive diagnostic and therapeutic effort with a unique peroperative enteroscopic approach.

## 1. Introduction

Bleeding from the varices of the gastrointestinal tract is a relatively common complication associated with long-term portal hypertension. This condition is associated with high mortality due to the usually intense blood loss [1]. The incidence of varices in other parts of the digestive tract is significantly lower. Bleeding from ectopic varices accounts for 1–5% of all variceal bleedings [2]. Varicose veins that are not located at predilection sites accessible by standard gastrofibroscopy or colonoscopy can cause a significant diagnostic problem. Ectopic varices have a four-fold higher risk of bleeding compared to esophageal varices and can have a mortality of up to 40% [3]. To date, several cases of ileal varices have been reported, but management and therapy have varied from case to case. This is caused by the absence of randomized studies to guide the management and treatment of such patients. We report a case of recurrent, hemodynamically significant bleeding from an ileal varix in a 63-year-old patient, which was diagnosed with an exploratory laparotomy using perioperative enteroscopy and treated by partial resection of the small intestine.

## 2. Case Presentation

**Figure 1:**
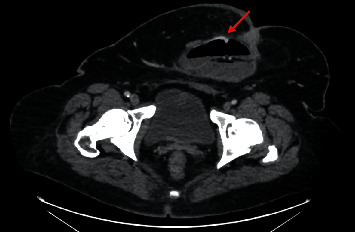
CT angiography showing a varix in the sac of the umbilical hernia.

**Figure 2:**
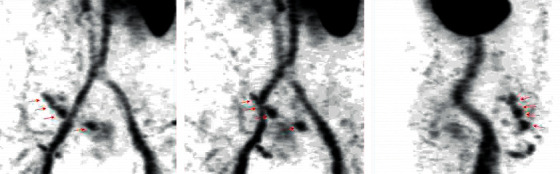
Red blood cell nuclear scan showing variceal vessels in the area of the hernia.

**Figure 3:**
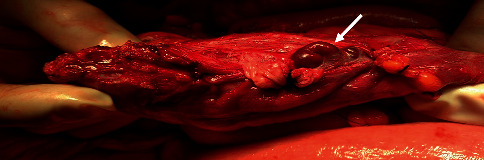
The ileum with the identified varix.

**Figure 4:**
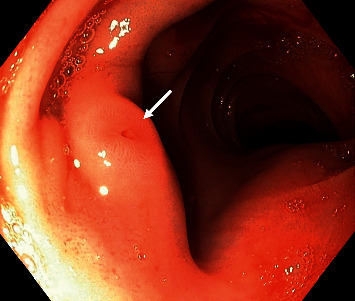
Enteroscopy showing the nipple sign at the site of the varix.

**Figure 5:**
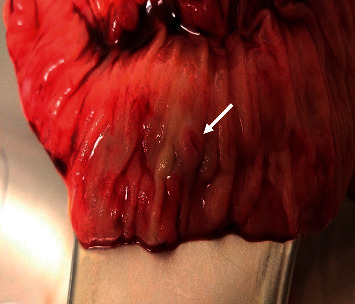
The resected part of the ileum.

**Figure 6:**
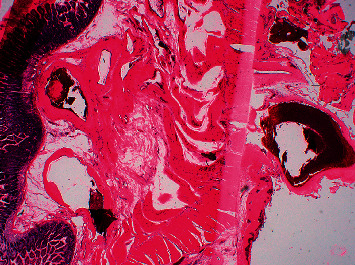
Histological examination of the resected part of the ileum.

We admitted to our department a 63-year-old patient with obesity grade 3 (BMI = 53.36 kg/m^2^), type 2 diabetes mellitus, and liver cirrhosis (Child–Pugh B) caused by nonalcoholic steatohepatitis because of gastrointestinal bleeding with a high risk of needing interventions and transfusions (Glasgow–Blatchford score 4 points). One year before, the patient was examined for anemia identifying diverticulosis as the source of bleeding. In the past, the patient also underwent an open surgery of the right ovary, cholecystectomy, and a hernioplasty but without the complete resolution of the umbilical hernia. Splenomegaly was also present. We proceeded by performing gastroscopy and colonoscopy without finding a source of bleeding but with obvious signs of gastrointestinal origin of the anemia as digested blood was present in the ileum and colon. The patient required repeated high amounts of transfusions of erythrocytes, and we were unable to stop the bleeding by conservative treatment. A control gastroscopy ruled out the esophagus, stomach, and duodenum as the site of bleeding. The jejunum was examined by single balloon-assisted enteroscopy. However, we could not reach the ileum as the patient's habitus, and several adhesions prevented the safe advance of the endoscope. CT angiography and red blood cell nuclear scan were unable to identify any leak into the gastrointestinal system but described variceal vessels in the sac of the umbilical hernia and perigastric and perisplenic area (shown in Figures 1 and 2). The patient's state progressed, and as all endoscopic and imaging methods were unsuccessful, we carried out an explorative laparotomy to find a 5 mm thick varix in the ileum without active bleeding during the time of surgery (Figure 3). We confirmed the varix as the culprit lesion by peroperative enteroscopy showing typical signs of recent bleeding (Figure 4). A resection of the affected part of the ileum was performed without significant early complications (Figure 5). The histological examination found erosions of the mucous membrane with edema and variceal vessels in the submucosal and subserosal layers (Figure 6). The patient was, after the operation, stabilized without any signs of bleeding. Four months after the surgery, a wound dehiscence occurred, needing further treatment.

## 3. Discussion

Massive bleeding from an ileal varix is a rare but serious condition with high mortality rates. The mechanism of the formation of ectopic varices is not entirely understood. Several risk factors for incidence are recognized, e.g., liver cirrhosis, portal hypertension, primary biliary cholangitis, hepatic vein thrombosis, chronic intraperitoneal inflammation, past surgery, and intraabdominal adhesions. The most common ileal shunting occurs through the gonadal veins. Hence, a past intervention in the lesser pelvis is another recognized factor. Currently, no guidelines for the treatment of ileal variceal bleeding are available as we still lack any randomized clinical studies concerning this condition. The diagnostic and therapeutical approach is derived from case reports and small retrospective studies [3]. Minowa et al. in 2017 summarized 21 published cases of ileal variceal bleeding. The most common diagnostic procedure was a form of angiography or multidetector computed tomography [4]. In 3 cases, capsule endoscopy was the diagnostic tool, and an enteroscopy was used just in one case [5, 6]. Abdominal surgery with the resection of the affected part of the intestine was a general approach [5]. We present a patient where a combined surgical and endoscopic management was realized as other methods of diagnosis and treatment failed in a timely fashion. Our patient presented with several risk factors of ileal variceal bleeding, as mentioned before. An essential factor that led to diagnostic uncertainties was the intermittent nature of the bleeding. Therefore, no leak of contrast on a CT scan or marked corpuscles on the red blood cell nuclear scan was recognized, even though these could diagnose the varix by itself. As bleeding did not occur even at the time of the surgery, we needed to ensure that the varix was the culprit lesion. Consequently, an intraoperative enteroscopy through a small incision in the ileum was performed with a typical finding of recent bleeding at the site of the varix. In the end, the affected part of the intestine and the place of insertion of the endoscope were resected. The whole procedure was being complicated by extreme obesity and comorbidities, nevertheless, it went uneventful. In the late postoperative period, dehiscence at the incision site of the abdominal wall evolved. After the surgery, the bleeding resolved, and an increase in the hemoglobin concentration was documented. Nonselective beta-blockers were introduced in our patient in the postoperative period but had to be withdrawn as hypotension evolved. TIPS is another recognized treatment choice but with a relatively high recurrence rate of bleeding [7]. We did not carry out the TIPS procedure during the acute state as we were not certain of variceal bleeding at the time. Capsule endoscopy was considered during the whole diagnostic process, but the limited availability of the examination and the worsening state of the patient precluded the execution. The risk of rebleeding after an acute episode of variceal bleeding is around 60%, but this data is mainly derived from esophageal variceal bleeding studies [8]. It can be theorized that even though the ileal varix was resected, portal hypertension still drives the process of varix development, and future variceal bleeding should be anticipated.

## 4. Conclusion

We present a case of ectopic variceal bleeding, which after extensive diagnostic effort, required an explorative laparotomy with peroperative enteroscopy to confirm the diagnosis. Resection of the affected part of the ileum was the definite treatment with a satisfactory outcome.

## Data Availability

No data were used to support this study.
